# EFFECTS OF THE COVID-19 ON MEDICAL USE OF ELDERLY PATIENTS WITH HYPERTENSION: A NATIONWIDE COHORT STUDY IN KOREA.

**DOI:** 10.1093/geroni/igad104.3701

**Published:** 2023-12-21

**Authors:** Eunbyul Cho

**Affiliations:** Seoul National University of Bundang Hospital, Seongnam, Gyeonggi, Republic of Korea

## Abstract

COVID-19 pandemic disrupted healthcare services, including chronic disease management for vulnerable groups like older individuals with hypertension. This study aims to evaluate hypertension management in South Korea’s elderly population during the pandemic using treatment consistency indices like Continuity of Care (COC), Modified, Modified Continuity Index (MMCI), Most Frequent Provider Continuity (MFPC). This study used the the Korea Disease Control and Prevention Agency-COVID-19-National Health Insurance Service cohort (K-COV-N cohort) from the National Health Insurance Service (NHIS) between 2017 and 2021 The research included a total of 4,097,299 hypertensive patients aged 65 or older. We defined 2018 and 2019 as baseline period before COVID 19 pandemic, and 2020 and 2021 as COVID 19 period, and calculated the indices of medical continuity (Number of visits, COC, MMCI and MFPC) on a yearly basis were measured. The number of visits were decreased during COVID 19 period compared to baseline period ( 59.64 ±52.75 to 50.49 ± 50.33, p< 0.001) respectively. However COC, MMCI and MFPC were not decreased in baseline period compared to COVID-19 period ( 0.71 ± 0.21 vs. 0.71 ± 0.22, p< 0.001), (0.97 ± 0.05 vs. 0.96 ± 0.05, p< 0.001), and (0.8 ± 0.17 vs. 0.8 ± 0.17, p< 0.001) respectively. In conclusion, COVID-19 had no significant impact on continuity of care, but it did affect the frequency of outpatient visits of older hypertensive patients. However, this study highlights the importance of addressing healthcare inequalities, especially for older hypertensive patients, during pandemics and advocates for policy changes to ensure continued care for vulnerable populations.

